# Preventing pneumococcal infections in patients with hematological malignancies: a review of evidence and recommendations based on modified Delphi consensus

**DOI:** 10.3389/fonc.2025.1546641

**Published:** 2025-05-01

**Authors:** Tulika Seth, Sameer Melinkeri, Tuphan Kanti Dolai, Jina Bhattacharyya, Neeraj Sidharthan, Prantar Chakrabarti, Chaithanya Malalur, Santosh Taur

**Affiliations:** ^1^ Department of Hematology, All India Institute of Medical Sciences (AIIMS), New Delhi, India; ^2^ Department of Hematology, Deenanath Mangeshkar Hospital, Pune, India; ^3^ Department of Hematology, NRS Medical College and Hospital, Kolkata, India; ^4^ Department of Hematology, Gauhati Medical College and Hospital (GMCH), Guwahati, India; ^5^ Department of Hematology, Amrita Hospital, Kochi, India; ^6^ Consultant Hematologist, ZOHO Corporation, Tenkasi, India; ^7^ Department of Medical Affairs, Pfizer Ltd., Mumbai, India

**Keywords:** hematological malignancies, hematopoietic stem cell transplant, pneumococcal Infections, risk, vaccines, consensus, delphi

## Abstract

**Introduction:**

Individuals with hematological malignancies (HMs) are at a high risk of invasive pneumococcal disease due to underlying malignancy and subsequent immunosuppressive anticancer therapy. Early management of pneumococcal infections is crucial for reducing morbidity and mortality in this vulnerable patient subgroup. In this study, we aim to review the current evidence and recommendations regarding the use of pneumococcal conjugate vaccines (PCVs) in patients with HMs and develop a consensus document on the optimal timing and patient profiles who can benefit from them.

**Methods:**

The modified Delphi consensus method was used for achieving consensus. The panel comprised a scientific committee of six experts from India. Questions were drafted for discussion around: (i) the risk and consequences of pneumococcal disease in HMs; (ii) barriers to pneumococcal vaccination in the hemato-oncology clinical setting; and (iii) evidence and optimal timing of pneumococcal vaccines in HMs. The questionnaire was shared with the panel through an online survey platform (Delphi round 1). The consensus level was classified as high (≥80%), moderate (60%–79%), and low (< 60%). A Delphi round 2 meeting was conducted to discuss the questions that received near or no consensus to reach an agreement. The final draft of consensus statements was circulated among the experts for approval.

**Results:**

Pneumonia with or without bacteremia and bacteremia without foci of infection are the most frequently reported clinical presentations of pneumococcal infections in patients with HMs. A high risk of pneumococcal disease has been observed in patients with multiple myeloma (MM), acute myeloid leukemia (AML), acute lymphoblastic leukemia (ALL), and chronic lymphocytic leukemia (CLL). Priming with PCV enhances the response to pneumococcal polysaccharide vaccine 23 (PPSV23) in patients with HMs. Experts agreed that PCV is beneficial and can be strongly recommended in patients with CLL, MM, and patients undergoing hematopoietic stem cell transplantation. Children with acute lymphoblastic leukemia (ALL) would benefit from systematic revaccination with PCV after chemotherapy. The evidence is inadequate to consistently recommend pneumococcal vaccination to all patients with lymphoma, AML, and adults with ALL.

**Conclusion:**

This expert consensus will guide clinicians on the recommended approach for administering pneumococcal vaccination to patients with HMs.

## 1 Introduction

**Figure 1:**
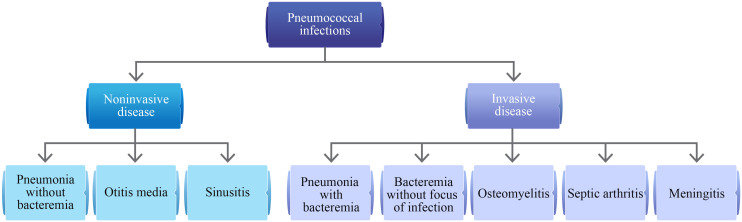
Clinical spectrum of pneumococcal infections (1).

Globally, pneumococcal diseases are a common cause of morbidity and mortality. The clinical spectrum of pneumococcal diseases varies from mild, noninvasive infections to severe, invasive infections (**Figure 1**) (1). *Streptococcus pneumoniae* can cause severe and potentially fatal diseases, notably pneumonia, bacteremia, and meningitis (1). Patients with hematological malignancies (HMs) have a high risk of invasive pneumococcal disease (IPD) due to underlying cancer and immunosuppressive anticancer treatments that are frequently associated with prolonged neutropenia and bone marrow suppression (2–5). These conditions usually increase the risk of serious infections requiring hospitalization (5). According to data from the Centers for Disease Control and Prevention, the rate of IPD was 129 per 100,000 population (during the period of 2013–2014) in adults (aged 18–64 years) with hematological cancer (1). The risk of IPD may vary by age, type of HM, and the time elapsed since the cancer diagnosis (2–4). Among different kinds of HMs, an increased risk of IPD was reported in patients with multiple myeloma (MM), acute lymphoblastic leukemia (ALL), and chronic lymphocytic leukemia (CLL) (2, 4). This increased susceptibility highlights the critical need for effective preventive measures, including vaccination. Anderson MA et al. reported a 3.5% annual decline in the incidence of IPD among adults following the introduction of pneumococcal vaccination in childhood vaccination programs (2). The reduction was even more pronounced in individuals with HM, showing a 9% annual decrease (2). Although pneumococcal infections are vaccine-preventable, this might often be overlooked by clinicians and patients with cancer as they may prioritize treating the cancer over vaccinating against the infection (6). Results of the global, prospective INSIGHT MM study conducted in patients with MM showed that pneumococcal vaccination in the previous 5 years impacted overall survival vs. no vaccination (p<0.0001). Furthermore, the proportion of deaths due to infections was lower among patients who were vaccinated than those not vaccinated (7). However, overall vaccination rates were low (30.2%) and varied by region, with the highest rates reported in the United States (42.83%) and the lowest in Asia (4.7%) (7). This discrepancy highlights a global challenge that needs addressing through tailored strategies and increased awareness.

In India, the situation is particularly concerning. Although pneumococcal vaccines (PVs) are available, vaccine uptake among patients with cancer in clinical practice is suboptimal. For example, a cross-sectional observational study conducted at the Tata Memorial Hospital between 2020 and 2023 found that only 0.68% of elderly patients with cancer (age ≥60 years) had received PV (8). Another study reported a vaccination prevalence of just 1.8% among adults with cancer (age ≥45 years) between 2017 and 2018 (9). These findings reveal the need for more rigorous measures and the adoption of specific recommendations and guidelines to improve the PV uptake in oncology clinical practice in India. Moreover, due to the paucity of well-designed randomized controlled trials (RCTs) conducted in India, hemato-oncologists rely on data from Western countries. In this study, we aim to review the current evidence and recommendations regarding the use of pneumococcal conjugate vaccines (PCVs) in patients with HMs and develop a consensus statement on the optimal timing and patient profiles who can benefit from them. We would also discuss barriers to pneumococcal vaccination in the hemato-oncology clinical setting and the recommended approach for administering pneumococcal vaccination to patients with HMs.

## 2 Methodology

### 2.1 Panel selection

**Figure 2:**
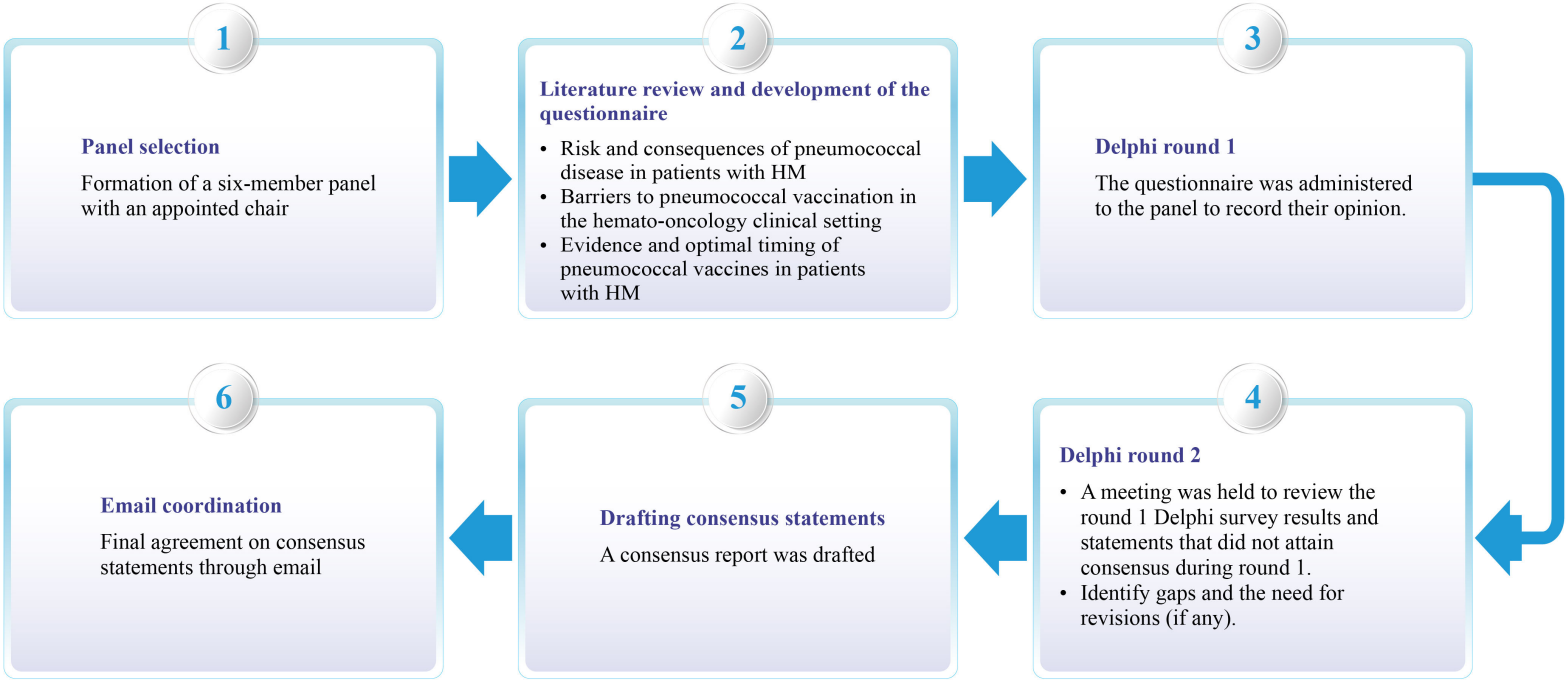
Process overview used to create the clinical consensus statement. HM, Hematological malignancy.

A panel comprising six experts (mean age: 52.17 years; specialty: hematology) was formed based on their academic record, clinical research involvement, and experience in managing hematological diseases from all four zones of India (North, South, East, and West). The experts were required to have at least 10 years of clinical expertise in the field. A chair was identified among the panel to moderate the consensus process (**Figure 2**).

### 2.2 Literature review and questionnaire development

A comprehensive literature search was conducted on the PubMed/MEDLINE database to identify pertinent articles published from January 1970 to March 2024. Diverse keyword combinations, including “burden”, “risk”,” hematologic malignancies”, “lymphoma”, “leukemia”, “multiple myeloma”, “immunocompromising”, “pneumococcal infections”, “pneumococcal pneumonia”, “invasive pneumococcal disease”, “pneumococcal conjugate vaccine”, “pneumococcal vaccine”, “efficacy”, “safety”, “pediatric”, “adult”, “prevention”, “guidelines”, and “management” were utilized. Variations in search phrases were applied, and Boolean operators (AND, OR) were used. The included sources comprised original research articles (RCTs, longitudinal studies, prospective and retrospective cohort studies, observational studies, case-control studies, and cross-sectional studies), systematic reviews, meta-analyses, practice guidelines, consensus recommendations, reviews, and surveys. Excluded sources were research studies involving animals or those published in a language other than English. Replicates were eliminated in the course of the filtering process. The questionnaire included relevant questions/statements under the following categories: (i) the risk and consequences of pneumococcal disease in patients with HM; (ii) barriers to pneumococcal vaccination in the hemato-oncology clinical setting; and (iii) evidence and optimal timing of PV in patients with HM. The questionnaire was finalized after discussions with the chair. Key articles were shortlisted and shared with the participants before the survey. The questionnaire was then rolled out through an electronic survey link to all the participants through an online survey platform (Delphi round 1).

### 2.3 Consensus process

A modified Delphi consensus method was used to achieve consensus (10). The level of consensus (**Table 1A**) was classified into three categories: (i) high (≥80%), moderate (60%–79%), and low consensus (<60%) (11). The expert participants discussed the survey results in the virtual meeting (Delphi round 2) on 9 March 2024. During the meeting, the experts discussed the questions receiving near or no consensus, discussed any differences in opinions, and modified the statements accordingly. Experts arrived at decisions based on available evidence and their current clinical practice. **Table 1B** lists the grades of recommendation (GOR) used during electronic voting (12). The consensus statements and recommendations were circulated to the experts for review. Another round of basic literature search was performed in PubMed/MEDLINE in July 2024 to check for any new updates. In the first week of July 2024, the final draft of the consensus statements and recommendations was circulated to the experts for their final review and approval.

**Table 1A:** Level of consensus (11).

Level of consensus	
“High”	“When ≥80% of participants agree/strongly agree or disagree/strongly disagree with a statement”.
“Moderate”	“When 60%–79% of participants agree/strongly agree or disagree/strongly disagree with a statement”.
“Low”	“When <60% of participants agree/strongly agree or disagree/strongly disagree with a statement”.

Adapted from: Jünger S et al., 2012 (11).

**Table 1B:** Grades of recommendation (12).

Grade of recommendation	
++	“This investigation or therapeutic intervention is highly beneficial for patients, can be recommended without restriction and should be performed”.
+	“This investigation or therapeutic intervention is of limited benefit to patients and can be performed”.
+/−	“This investigation or therapeutic intervention has not shown benefit for patients and may be performed only in individual cases. According to current knowledge, a general recommendation cannot be given”.
−	“This investigation or therapeutic intervention can be of disadvantage to patients and might not be performed”.
−−	“This investigation or therapeutic intervention is of clear disadvantage to patients and should be avoided or omitted in any case”.

Adapted from: Scharl A et al., 2013 (12).

## 3 Results

### 3.1 Risk and consequences of pneumococcal infections in patients with HMs

Patients with HMs and lymphoid malignancies are at an increased risk for IPD compared with the general population (2–4, 13). *Streptococcus pneumoniae* is the predominant cause of IPD and community-acquired pneumonia (CAP) in Indian adolescents and adults (14, 15). Studies report that the case fatality rate (CFR) for IPD in India ranges from 17.8% to 30% (15, 16). A population-based surveillance study (1995–2012) for IPD conducted in Canada reported CFRs of 11.8% and 22.4% in patients with IPD and HMs aged 15–64 years and ≥65 years, respectively (17). In a population-based cohort study conducted in the Netherlands from 2004 to 2016, the IPD incidence rate was 482 of 100,000 in adults with HMs vs. 15 of 100,000 without a malignancy (3). The hospital intensive care unit admission rate was 15.1% in patients with HMs and IPD. The IPD-related CFR was higher in patients with HMs vs. those without malignancies, and an increase in IPD-related CFR with age was noted (3). Another population-based cohort study (2000–2016) by Andersen MA et al. reported similar findings, with IPD incidence rates of 421.1 per 100,000 person-years in individuals with HMs vs. 12.7 per 100,000 person-years in those without HMs in Denmark (2). The study highlighted that the IPD incidence rate was highest for individuals with MM, ALL, and CLL. Incidence rates were lowest for Hodgkin lymphoma (2). Patients with chronic myeloid leukemia (CML) are usually not considered at high risk of infection unless they develop neutropenia or acute transformation. Studies mention that CML patients have an increased risk of respiratory and skin infections (pneumonia, sinusitis, bronchitis, and cellulitis) vs. healthy individuals (18, 19).

In patients with HMs, pneumococcal disease is associated with complications resulting in hospitalizations and other morbidities (1, 3–5, 7, 17, 20). Complications of pneumococcal pneumonia include bacteremia, empyema, pericarditis, and endobronchial obstruction, with atelectasis and lung abscess formation (1). Furthermore, pneumococcal bacteremia can occur with or without pneumonia, leading to arthritis, meningitis, and endocarditis (1). Chen CL et al. found that pneumonia was the primary reason for admission to the intensive care unit (ICU) among patients with HMs, accounting for 45.1% of cases, followed by septic shock (25.8%) (20). Moreover, patients with HMs in the ICU face high mortality rates, often predicted by the need for mechanical ventilation and vasopressor therapy (20–22). The vulnerability of this population makes these infections particularly significant. Early identification and prompt management of pneumococcal infections is crucial for reducing morbidity and mortality in this high-risk patient subgroup.

#### 3.1.1 Expert opinions

Pneumonia with or without bacteremia and bacteremia without foci of infection are the most frequently reported clinical presentations of pneumococcal infections in Indian patients with HMs. On the other hand, meningitis, acute otitis media, and sinusitis are less commonly reported. Experts agreed that in patients with HMs, a majority of pneumococcal infections do not resolve in less than 2 weeks (100%), require prolonged treatment (83.3%), carry a high risk of complications (83.3%), and pose a significant mortality risk (100%). Four out of six (66.7%) experts concurred that most patients with HMs at their institutions required emergency hospitalization to manage IPD. Experts mentioned that 15%–30% of their patients with HMs required admission to intensive care units. Among Indian patients with HMs, a high risk of pneumococcal disease has been observed in patients with MM, acute myeloid leukemia (AML), ALL, and CLL. On the other hand, the risk is lower in patients with CML, Hodgkin, and non-Hodgkin lymphoma (NHL).

### 3.2 Evidence and optimal timing of pneumococcal vaccinations in patients with HMs

Studies suggest that the introduction of routine childhood pneumococcal vaccination programs may have an indirect effect on IPD incidence rates in patients with HMs (2, 3, 17, 23). Two population-based cohort studies showed a 9% annual decrease in IPD incidence among adult patients with HMs coinciding with the introduction of PCV7 and PCV13 and a significant 35% decline following the introduction of PCV7 and PCV10 (2, 3). A retrospective, longitudinal cohort study showed a significant 64% decline in the incidence of IPD after vaccination with PCV7 and a nonsignificant decline of 46% in the same with PCV13 vaccination in patients with HMs (23). Shigayeva A, et al. reported a significant decline in the incidence of IPD in immunocompromised adults after the introduction of pneumococcal polysaccharide vaccine 23 (PPSV23) and PCV7 vaccination programs (incidence rate ratio=0.57) (17). Routine pneumococcal vaccination as part of infection prophylaxis may offer protection against adverse outcomes in patients with HMs. Results from the INSIGHT MM study in adult patients with newly diagnosed (ND) or relapsed/refractory MM showed that pneumococcal vaccination in the prior 5 years vs. no vaccination affected overall survival (p<0.0001) (7). The proportion of infection-related deaths was significantly lower among vaccinated vs. unvaccinated patients (9.9% vs. 18.0%; p=0.032) (7). A retrospective study by Draliuk R et al, highlighted that adult patients with HMs vaccinated with PCV13 before initiating immunosuppressive therapy had significantly reduced odds of hospitalization due to pneumonia or sepsis vs. those not vaccinated (p=0.012) (5). The ability of conjugated PVs to prime for booster responses was shown in a study evaluating the effects of priming with PCV7 on the antibody responses to PPSV23 in previously treated patients with Hodgkin disease (HD) (24). Patients receiving PCV7 followed by PPSV23 showed significantly higher averaged antibody geometric mean concentrations across the six common serotypes vs. those receiving only PPSV23 (12.5 vs. 7.76 µg/mL; p=0.015). These findings supported that prior PCV immunization effectively primes for later responses to PPSV23 and may reduce the number of vaccine failures (24).

#### 3.2.1 Pneumococcal vaccinations in patients with cancer, including HMs

Two PCV7 doses elicited protective antibody titers in 86%–100% of pediatric patients with cancer who had discontinued chemotherapy (ChT) 3–12 months before vaccination or were on maintenance ChT for ALL (25). Similarly, single-dose PCV13 elicited protective antibody titers for most serotypes in ≥70% of children with cancer who were either receiving or within 12 months of completing immunosuppressive therapy (26). Single-dose PCV13 administered to pediatric patients with cancer or three PCV13 doses ~4 weeks apart given to adult patients elicited elevated pneumococcal immunoglobulin G (IgG) and opsonophagocytic activities (OPAs) that showed persistent protection for 6 months (27). Across the studies, minimal or no serious adverse events (SAEs) were noted (25–27).

#### 3.2.2 Pneumococcal vaccinations in patients with MM

PPSV23 elicited protective antibodies in 40% of patients with MM (28). In a retrospective study by Hinge M, PPSV23 given before autologous stem cell transplantation produced responses in 33% of patients with MM, with better responses in those with good disease control (**Table 2**) (29–34). There were no reports of serious adverse reactions to vaccination (28, 29). Mustafa SS et al. reported that patients with MM showed similar initial response rates to PCV13 vs. controls but with a significant drop in response over time (30). Similarly, in a study largely including ND patients who received PCV13 followed by PPSV23, 85% showed responses to ≥1 antibody subtype, but there was a decrease in antibody concentrations over time (31). In patients with relapsed myeloma, two PCV7 doses given concomitantly with lenalidomide produced better responses vs. giving one dose before starting lenalidomide and the second dose while on lenalidomide (32). In a retrospective study by Palazzo M et al., a three-dose PCV13 regimen initiated at ~1 year after hematopoietic stem cell transplantation (HSCT) showed good response rates (58%), not affected by lenalidomide maintenance, in patients with MM (33). In patients with MM receiving novel agents (bortezomib, lenalidomide, ixazomib), a three-dose PCV13 regimen was associated with a 33.3% absolute risk reduction in pneumonia compared with no vaccination (34). There were no vaccine-related adverse effects (33, 34).

**Table 2:** Response and safety of pneumococcal vaccinations in patients with MM (29–34).

Author and year	Study design and patient population	Type of PV administered	Key results
Hinge M et al., 2012 (29)	Retrospective study Patients with MM (N=60)	A single dose of PPSV23 before autologous SCT	• Responses in 33% of patients • Significant association was noted between response and disease stage (p=0.01): ᠅ CR stage: 73% responded ᠅ PR stage: 25% responded ᠅ MR stage: 17% responded ᠅ NR stage: None responded • No serious adverse reaction to the vaccine
Mustafa SS et al., 2019 (30)	Prospective cohort study Patients with MM (N=7) Normal controls (N=18)	PCV13 42.9% of patients were receiving ChT during the study	• Immediate response: No difference between MM patients vs. controls • Durability of response at 6 months: 1 of 3 patients with MM vs. 7 of 7 controls (p=0.02)
Renaud L et al., 2019 (31)	Prospective study Patients with MM (N=28), of whom 25 were ND, 2 at first relapse, and 1 at second relapse	PCV13 and then PPSV23 Patients were allowed to receive induction ChT in between the two vaccinations.	• Response to ≥1 antibody subtype in 85% of patients, ≥2 subtypes in 65%, ≥3 subtypes in 55%, and all four subtypes in two patients • Decrease in the antibody GMC over time for all subtypes
Noonan K et al., 2012 (32)	Early phase, open-label, two-cohort study Patients with relapsed myeloma following 1–3 prior therapies, but lenalidomide-naïve (N=22)	Cohort A: First PCV7 dose 2 weeks before initiating lenalidomide. Second PCV7 dose during lenalidomide treatment (n=11) Cohort B: Both PCV7 doses during lenalidomide treatment (n=11)	• Cohort A: Stable or decreased antibody titers • Cohort B: Continuous increase in antibody titers • PCV-specific T-cell responses are greater in Cohort B than in Cohort A
Palazzo M et al., 2018 (33)	Retrospective Patients with MM on lenalidomide maintenance after autologous HSCT (N=119)	3Ds of PCV13 at 1–3-month intervals starting at ~1 year after HSCT	Response to PCV13 series: • Response is shown in 58% of patients • No difference in response rates for those receiving vs. not receiving lenalidomide maintenance Safety: • No vaccine-related adverse effects
Stoma I et al., 2020 (34)	Prospective, non blinded, randomized study Patients with MM receiving novel agents (N=36): Vaccinated group (n=18) and unvaccinated matched controls (n=18)	3Ds of PCV13 with a minimum of 1-month interval between treatment courses with novel agents	• Confirmed pneumonia (vaccinated group: 16.7%; unvaccinated group: 50%; p=0.037) • 33.3% absolute risk reduction of pneumonia in patients receiving PCV13 • No fatal outcomes were associated with pneumonia • No adverse effects of vaccination

3Ds, Three doses; ChT, Chemotherapy; CR, Complete response; GMC, Geometric mean concentration; HSCT, Hematopoietic stem cell transplantation; MM, Multiple myeloma; MR, Minimal response; ND, Newly diagnosed; NR, No response; PCV, Pneumococcal conjugate vaccine; PPSV, Pneumococcal polysaccharide vaccine; PR, Partial response; PV, Pneumococcal vaccine; SCT, Stem cell transplantation.

#### 3.2.3 Pneumococcal vaccinations in ALL and AML

In children with ALL, PCV13 during maintenance ChT elicited suboptimal responses, whereas vaccination at either 4 weeks or 6 months after maintenance elicited comparable protective immunity (35). Dorval S reported that PCV13 administered during maintenance ChT conferred modest seroprotection at the end of ChT. However, a dose after ChT was necessary and sufficient to attain high seroprotection (to *S. pneumoniae*) (36). Top et al. showed that PCV13 given 4–12 months after ChT elicited good seroprotective IgG levels (37). There were limited or no reports of vaccine-related SAEs (**Supplementary Table S1**) (35–37). There is a lack of robust evidence for the immune responses to pneumococcal vaccinations in exclusive AML patient populations. Patel SR et al. showed that children with AML or ALL did not have protective antibody levels against all PCV7 and additional PCV13 serotypes at 6 months after ChT (38). The results suggested that patients with AML may benefit from PCV13 vaccination at ~6 months after completing ChT (38).

#### 3.2.4 Pneumococcal vaccinations in patients with CLL and CML

Suboptimal antibody responses to PPSV23 were noted in patients with CLL (39–41). However, responses were better in patients with a less advanced disease stage (**Table 3**) (39, 42–46). Single-dose PCV7 elicited lower antibody responses in patients with CLL vs. controls. However, significant responses were noted in ~40% of patients if the vaccine had been given at an early disease stage (42). A follow-up study showed persistent antibody responses to PCV7 for at least 5 years, with protective antibody levels for most serotypes in >50% of patients (47). Single-dose PCV13 induced an adequate response in 58% of treatment-naïve patients with CLL. Responders had lower clinical stages of CLL (43). In a prospective study by Mauro FR et al., single-dose PCV13 elicited adequate responses in nine patients (8%), of whom eight were treatment-naïve (44). No responses to PCV13 were noted in four patients receiving ibrutinib (45). PCV13 elicited a better immune response vs. PPSV23 at 1 and 6 months after vaccination in treatment-naïve patients (45). Svensson T et al. highlighted a longer disease burden was a negative predictor of vaccination response and PCV13 should be administered as early as possible in treatment naïve patients with CLL (46). Across studies, no serious PV-related side effects were noted in patients with CLL (39, 43, 44, 46). Patients with CML receiving tyrosine kinase inhibitors showed suboptimal immunoglobulin M humoral response to PPSV23 compared with healthy controls (48).

**Table 3:** Response and safety of pneumococcal vaccinations in patients with CLL (39, 42–46).

Author and year	Study design and patient population	Type of PV administered	Key results
Hartkamp A et al., 2001 (39)	Patients with CLL (N=25)	PPSV23	Antibody responses: • Protective antibody levels in 50% of patients • Characteristics of responders: Less advanced disease stage, ChT-naïve, higher gamma globulin, total IgG, IgG2, and IgG4, and lower-soluble CD23 Safety: • No major local or systemic reactions
Sinisalo M et al., 2007 (42)	Prospective trial Patients with CLL (N=52) Matched controls without any immunological or hematological defect (N=25)	Single dose of PCV7	Antibody responses: • A significant response in 20%–47% of patients with CLL vs. 75%–88% of controls • Significant response to ≥6 antigens in 39% of patients if the vaccine had been given at an early disease stage
Pasiarski M et al., 2014 (43)	Treatment-naïve patients with CLL (N=24) Healthy controls (N=15)	A single dose of PCV13	Antibody responses: • Adequate response in 58.3% of patients vs. 100% of controls • Characteristics of responders: Lower CLL clinical stage, higher total IgG and IgG2 Safety: • No major side effects related to vaccination
Mauro FR et al., 2021 (44)	Prospective study Patients with CLL (N=112) who were either treatment-naïve (n=22) or previously treated (n=90)	Single dose of PCV13	Immune response: • Adequate immune response in nine patients (8%), of whom eight were treatment-naïve • Factors associated with lower responses: Age ≥60 years, baseline IgG <400 mg/dL, prior treatment, and signs of disease progression Safety: • Vaccine was well tolerated
Andrick B et al., 2018 (45)	Prospective, nonblinded study Patients with CLL	Single dose of PCV13 in two study cohorts: • No active treatment for CLL (control cohort) • Active treatment with ibrutinib	Adequate response in all controls vs. none of the patients on ibrutinib (p=0.029)
Svensson T et al., 2018 (46)	Randomized, nonblinded trial Treatment-naïve patients with CLL (N=128)	PCV13 (n=63) or PPSV23 (n=65)	Immune response: • Positive immunological response in more PCV13 than in PPSV23 recipients after 1 month (p=0.034) and after 6 months (p=0.041) Safety: • No vaccine-related SAEs

CLL, Chronic lymphocytic leukemia; ChT, Chemotherapy; IgG, Immunoglobulin G; PCV, Pneumococcal conjugate vaccine; PPSV, Pneumococcal polysaccharide vaccine; PV, Pneumococcal vaccine; SAE, Serious adverse event.

#### 3.2.5 Pneumococcal vaccinations in patients with lymphoma

Splenectomized patients with HD or NHL showed good antibody responses to PPSV administered before splenectomy and treatment; however, nonresponder patients with NHL did not benefit from revaccination (49, 50). Significant responses to primary PPSV23 vaccination and two revaccinations were noted in splenectomized patients with HD (51). Cherif H et al. reported poor responses to PPSV23 in 28% of splenectomized patients with hematological disorders who did not benefit from revaccination (52). Overall, no severe adverse reactions to PPSV23 were noted (**Supplementary Table S2**) (49–54).

Previously treated patients with HD showed suboptimal responses to PCV7 vs. healthy controls or those receiving PPSV23 (53). Lee D et al. found that PCV13 given at 3 or 6 months after CD19-targeted chimeric antigen receptor-modified T cell therapy (CAR-T) did not induce humoral protection in patients with relapsed/refractory large B-cell lymphoma (54).

**Table 4** lists the optimal timing of pneumococcal vaccinations in patients with HMs and patients undergoing splenectomy (19, 55–65).

**Table 4:** Recommendations for the administration of pneumococcal vaccinations in patients with cancer, including HMs, (A) and patients undergoing splenectomy (B) (19, 55–65).

A) Cancer, Including HMs	
NCCN (55)	“PCV20 should be administered to adults who are ND with cancer and who are PV naïve. Alternatively, PCV15 can be given, followed by PPSV23 ≥8WL.”
ASCO (56)	“1D of PCV15 followed by PPSV23 8WL (in adults) or 1D of PCV20^a^ (in adults)”
CDC (57, 58)	Children aged 2–5 years with HMs, asplenia: • “Three prior PCV doses: 1D of PCV (≥8 weeks after the most recent dose)” • “<3 prior PCV doses: 2Ds of PCV (≥8 weeks after the most recent dose and given ≥8 weeks apart)” • “Not previously received PCV20: 1D of PCV20 or 1D of PPSV23 ≥8 weeks after the most recent PCV dose” Children aged 6–18 years with HMs, asplenia: • “No prior PCV or PPSV23: 1D of PCV15 or PCV20. If received PCV15 dose, then 1D of PPSV23 ≥8WL” • “Received PCV before the age of 6 years but not PPSV23: Not previously received PCV20, then administer 1D of PCV20 or PPSV23 dose 1 ≥8 weeks after PCV and then a second dose of PPSV23” • “Received PCV13 only at or after the age of 6 years: Administer 1D of PCV20 or PPSV23 dose 1 ≥8 weeks after PCV13 and then a second dose of PPSV23” “Note: Second dose of PPSV23 to be administered ≥5 years after PPSV23 dose 1” Adults 19–49 years: • “Not previously received a PCV13, PCV15, PCV20, or PCV21 or whose previous vaccination history is unknown: 1D of PCV21 or 1D of PCV20 or 1D of PCV15 followed by 1D of PPSV23 ≥8WL” • “Received only PCV13: 1D of PCV21 or 1D of PCV20 at least 1 year later” Adults ≥50 years old: • “Not previously received a PCV13, PCV15, PCV20, or PCV21 or whose previous vaccination history is unknown: 1D of PCV21 or 1D of PCV20 or 1D of PCV15 followed by 1D of PPSV23 ≥8WL” • “Received only PCV13: 1D of PCV21 or 1D of PCV20 at least 1 year later”
ICS–NCCP (59)	In adults with immunocompromising conditions^b^: • “19–64 years of age: 1D of PCV13 and PPSV23 ≥8WL” • “≥65 years of age: PCV13 first, followed by PPSV23 is recommended for individuals with immunocompromising conditions, functional or anatomic asplenia, or a history of IPD”
IMA (60)	In patients with cancer: • “PCV: 1D at not less than 3 months after cancer ChT” • “PPSV23: 1D ≥8 weeks after PCV”
IAP–ACVIP (61)	Children aged 24 through 71 months in high-risk conditions: • “Three prior PCV13 doses: 1D of PCV13 and 1D of PPSV23 after 8 weeks” • “<3 prior PCV13 doses: 2Ds of PCV13 ≥8 weeks apart, and PPSV23 ≥8WL”
ECIL (19)	• AML: “Administer PVs 3–6 months after ChT” • CML: “1D of PCV followed 2 months later by 1D of PPSV23” • MM: “1D of PCV13 followed by 1D of PPSV23 ≥8WL, preferably before treatment or during maintenance” • Lymphoma: “1D of PCV13 then 1D of PPSV23 ≥8WL, preferably before treatment or during maintenance, except in patients who are receiving high-dose ChT or who are receiving or have received anti-CD20 antibodies in the previous 6 months” • CLL: “1D of PCV13 followed by 1D of PPSV23 ≥8WL preferably before treatment” • Children with ALL: ◦ “During maintenance” ◦ “From ≥3 but preferably 6 months after ChT”
GSI (62)	“Recommends both PCV13 and PPSV23 for all individuals older than 50 years and immunocompromised individuals with a high risk for pneumococcal infections”. • “Consider PCV13 for elderly individuals who have previously received PPSV23” • “If previously unvaccinated, 1D of PCV13 then 1D of PPSV23 ≥8WL”.
IDSA (63)	• “PCV13 should be administered to ND adults with hematological or solid malignancies and children with malignancies. PPSV23 should be administered to adults and children (aged ≥2 years) ≥8 weeks after the indicated dose(s) of PCV13”. • “Vaccination timing after therapy (in patients with cancer): ≥3 months after cancer ChT and ≥6 months after regimens that include anti–B-cell antibodies”.
B) Patients Undergoing Splenectomy	
IAP (64)	“≥2 weeks before splenectomy”
AGIHO (65)	“Vaccinate 2 weeks before splenectomy at the latest or 14 days after surgery^c”^ “PCV13 first and then PPSV23 6–12 weeks later, revaccinate every 6 years”
IDSA (63)	“PPSV23^d^ ≥2 weeks before surgery (and following indicated dose[s] of PCV13) or ≥2 weeks after surgery”

a Patients who have previously received PCV13 can only receive 1D of PCV20 after 1 year.

b Include HMs, asplenia, or patients undergoing HSCT.

c If preoperative vaccination is not possible.

d For PPSV23-naïve patients aged ≥2 years for whom splenectomy is planned.

1D, One dose; 2D, Two doses; AGIHO, Infectious Diseases Working Party of the German Society for Hematology and Medical Oncology; ALL, Acute lymphoblastic leukemia; AML, Acute myeloid leukemia; ASCO, American Society of Clinical Oncology; CDC, Centers for Disease Control and Prevention; ChT, Chemotherapy; CLL, Chronic lymphocytic leukemia; CML, Chronic myeloid leukemia; ECIL, European Conference on Infections in Leukemia; 8WL, Eight weeks later; GSI, Geriatric Society of India; HM, Hematological malignancy; IAP, Indian Academy of Pediatrics; ICS-NCCP, Indian Chest Society and the National College of Chest Physicians of India; IDSA, Infectious Diseases Society of America; IMA, Indian Medical Association; NCCN, National Comprehensive Cancer Network; ND, Newly diagnosed; PCV, Pneumococcal conjugate vaccine; PPSV, Pneumococcal polysaccharide vaccine.

#### 3.2.6 Pneumococcal vaccinations in patients undergoing HSCT

Single-dose PPSV23 administered at a median of 756 days after allogeneic HSCT (allo-HSCT) showed positive OPA response rates of 55% at 1 year after vaccination in patients aged 20–70 years (**Table 5**) (66–79). In a retrospective study by Pao M, a three-dose PCV7 series from ~1 year after allo-HSCT showed response rates of 62%, with higher responses in children vs. adults (67). In pediatric patients, a three-dose PCV7 regimen starting 6–9 months after allo-HSCT elicited complete protection in 74% of patients (68). In the EBMT-IDWP01 trial, a three-dose PCV7 regimen starting at either 3 (early group) or 9 months (late group) after allo-HSCT elicited similar responses between the early and late groups, with a significant correlation between IgG and OPA titers for all PCV7 serotypes (80–82). In this trial, a dose of PPSV23 at either 12 or 18 months after HSCT improved the responses to PCV7 antigens, but this boost effect was higher in the late group vs. the early group. Regardless of the timing, the PPSV23 dose also broadened the pneumococcal serotype coverage (80–82). A 10-year follow-up of this trial revealed that antibody levels were well maintained at 8–11 years after transplant (69). Molrine DC et al. reported that among allo-HSCT recipients receiving PCV7 at 3, 6, and 12 months after transplant, those patients whose donors had received PCV7 before HSCT had improved antibody responses within the first year of HSCT (70). By 13 months, >60% of patients had protective antibody concentrations regardless of donor immunization (70). Similar findings were reported among autologous HSCT recipients receiving single-dose PCV7 before HSCT followed by the same three-dose PCV7 regimen after transplant (71). Kumar D et al., reported that in adult allo-HSCT recipients, donor immunization with PCV7 followed by recipient immunization at 6 months after HSCT showed greater responses vs. a similar strategy with PPSV23 (90.9% vs. 55.6%) (72).

**Table 5:** Response and safety of pneumococcal vaccinations in patients undergoing HSCT (66–79).

Author and year	Study design and patient population	Type of PV administered	Key results
Okinaka K et al., 2017 (66)	Prospective, open-label, single-arm study Allo-HSCT recipients aged 20–70 years with HM (N=30)	Single dose of PPSV23 (median time from allo-HSCT to vaccination was 756 days)	Median positive response rates after PPSV23 • By IgG: 43% at 1 month and 43% at 1 year • By OPA: 72% at 1 month and 55% at 1 year Safety • No severe adverse effects due to PPSV23
van der Velden AM et al., 2007 (77)	Prospective follow-up study Adult patients with NHL, MM, or amyloidosis who underwent autologous SCT (N=20)	Two PCV7 doses followed by a PPSV23 dose at 6, 8, and 14 months after transplant	Responses after the two PCV7 doses • Sufficient antibody responses in 33% of patients Response after the PPSV23 dose • Increased responders to conjugate serotypes (78%) Safety • No major adverse reactions after vaccination
Pao M et al., 2008 (67)	Retrospective study Pediatric and adult patients after an allo-HSCT (N=127)	Series of three PCV7 doses 4–8 weeks apart Median time to vaccination: ~1 year after HSCT	Vaccine response • Responses to PCV7 in 62% of patients (children vs. adults: 88% vs. 44%; p<0.001) Safety • No serious adverse reactions to vaccination
Meisel R et al., 2007 (68)	Prospective multicenter trial Pediatric patients up to 16 years of age recruited following allo-HSCT (N=53)	Three PCV7 doses in monthly intervals starting at 6–9 months after HSCT	Vaccine responses • Complete protection against all seven serotypes: 55.8% of patients after the second dose and 74.4% after the third dose Safety • Four SAEs were observed; none related to the vaccine
Cordonnier C et al., 2015 (69)	EBMT-IDWP01 trial (follow-up)	Three PCV7 doses given a month apart started at either ~3 months (early group) or ~9 months (late group) after HSCT A dose of PPSV23 at 12 months (early group) or 18 months (late group) after HSCT	Response rates of 40% (antibody cutoff of 0.50 μg/mL) vs. 65.5% (antibody cutoff of 0.15 μg/mL) for seven antigens of PCV7
Molrine DC et al., 2003 (70)	Randomized controlled study Patients aged ≥2 years scheduled to receive allo-HSCT for HM (N=96)	Immunized donor group: Patients and donors received a dose of PCV7 at ~7–10 days before HSCT Unimmunized donor group: Patients and donors did not receive PCV7 before HSCT All study patients: PCV7 at 3, 6, and 12 months after HSCT	Antibody responses after HSCT • More patients with protective IgG levels in the immunized vs. unimmunized donor groups after the first PCV7 dose (67% vs. 36%; p=0.05) • Protective IgG levels after the third PCV7 dose in >60% of patients in both groups Safety • PCV7 was well tolerated
Antin JH et al., 2005 (71)	Prospective randomized study Patients >2 years old with an HM and scheduled to receive autologous HSCT (N=61)	PCV7 were given 7–10 days before stem cell collection or no vaccination Participants were given PCV7 at 3, 6, and 12 months after the transplant	Antibody responses after HSCT • Higher antibody concentrations in patients given PCV7 before HSCT • At 13 months, >60% of patients had protective concentrations regardless of preharvest vaccination Safety • No SAE related to immunization
Kumar D et al., 2007 (72)	Randomized, double-blind, controlled trial Adult patients undergoing allo-HSCT and their donors (N=64 donor–recipient pairs)	Strategy 1 (n=32): PCV7 in donors at least 2 weeks before stem cell collection; PCV7 in recipients at 6 months after HSCT Strategy 2 (n=32): PPSV23 in donors at least 2 weeks before stem cell collection; PPSV23 for the recipients at 6 months after HSCT	Response to ≥1 serotype after HSCT • At 12 months (after recipient vaccination): 90.9% in the PCV7 group vs. 55.6% in the PPSV23 group (p=0.02) Infections • No IPD during the study period Safety • Vaccines were well tolerated
Langedijk AC et al., 2019 (73)	Patients receiving immunization after allo-HSCT (N=103)	Starting at 1 year after HSCT, three PCV13 doses followed by a single PPSV23 dose	PCV13 serotype-specific antibody responses (n=39) • Sufficient seroprotection in 85% of patients across all PCV13 serotypes
Cordonnier C et al., 2015 (74)	Prospective, multicenter, open-label study Patients aged ≥2 years with hematological disorders who had undergone allo-HSCT (N=251)	Starting at ~3–6 months after HSCT, three doses of PCV13 at 1-month intervals, a fourth dose 6 months later, and a dose of PPSV23 1 month later	Immunogenicity: Significant increases in GMFRs of IgG GMCs across all PCV13 serotypes from baseline to post-dose 3 (GMFR: 2.99–23.85)
Garcia Garrido HM et al., 2022 (75)	Prospective, multicenter cohort study Adult allo-HSCT recipients (N=89)	Starting at 4–6 months after HSCT, four doses of PCV13 (at T0, T1, T2, T8*) and a dose of PPSV23 (at T10*) *T refers to months from enrollment.	Seroprotection rates at 12 months • PCV13/PPSV23 combined serotypes (33%), PCV13 serotypes (67%), and PPSV23-exclusive serotypes (19.2%) • Treatment with immunosuppressive agents at baseline was associated with less overall seroprotection (OR=0.36) and protection against PCV13 serotypes (OR=0.33) Antibody concentrations over time • Significant increase in IgG concentrations over time for all 24 serotypes Safety • A total of 14 SAEs in 12 patients; none were vaccine-related • No IPD episodes during the study period
Okinaka K et al., 2023 (76)	Phase 2, multicenter, open-label, randomized controlled trial Patients aged ≥2 years with HM who had undergone allo-HSCT and did not have active GVHD (N=72)	3+1+1 group: four doses of PCV13 at 0, 1, 2, and 8 months and 1D of PPSV23 at 9 months 3+0+1 group: three doses of PCV13 at 0, 1, and 2 months and 1D of PPSV23 at 9 months	Response rate: • The overall IgG response rate at 5 months after PPSV23 did not differ between the two groups (100% vs. 93%, RR=1.07) Safety • No SAEs leading to study dropout • No IPD cases during follow-up
Robin C et al., 2020 (78)	Adult allo-HSCT recipients assessed at a median of 9.3 years after transplant (N=100)	PCV** • All patients received ≥1 dose • 86% of patients received ≥3 doses, of whom 11 patients received four doses PPSV23** • 94% of patients received ≥1 dose, of whom 57 patients received ≥2 doses **Different vaccination schedules according to the date of transplant.	• No difference in seroprotection rates between the different vaccination schedules
Roberts MB et al., 2020 (79)	Retrospective, observational study Adult (age ≥16 years) autologous HSCT and allo-HSCT recipients (800 patients with 842 HSCT events)	Pre-2010 group: PPSV23 at 12 and 24 months after HSCT followed by a 5-yearly PPSV23 booster Post-2010 group: three doses of PCV10 or PCV13 at 6, 8, and 10 months after HSCT and another PCV13 dose at 14 months if GVHD present. All patients received PPSV23 at 24 months	IPD episodes Significant reduction in IPD rates from the pre-2010 to post-2010 group.

Allo-HSCT, Allogeneic hematopoietic stem cell transplantation; GMC, Geometric mean concentration; GMFR, Geometric mean fold rise; GVHD, Graft-versus-host disease; HM, Hematological malignancy; HSCT, Hematopoietic stem cell transplantation; IgG, Immunoglobulin G; IPD, Invasive pneumococcal disease; MM, Multiple myeloma; NHL, Non-Hodgkin lymphoma; OPA, Opsonophagocytic activity; 1D, One dose; OR, Odds ratio; PCV, Pneumococcal conjugate vaccine; PPSV, Pneumococcal polysaccharide vaccine; RR, Relative risk; SAE, Serious adverse event; SCT, Stem cell transplantation.

A three-dose PCV13 regimen starting from 1 year after allo-HSCT followed by PPSV23 at 18 months yielded response rates of 85% to PCV13 serotypes and 62% to PPSV23-only serotypes. However, ~43% of the IPD cases occurred in the first year after transplantation (73). Following a three-dose PCV13 series starting at 3–6 months after allo-HSCT, a fourth dose given 6 months later led to significantly increased antibody levels (74). A PPSV23 dose 1 month after the fourth PCV13 dose did not further increase responses to PCV13 serotypes (74). A similar four-dose PCV13 regimen starting at 4–6 months after allo-HSCT in adults followed by PPSV23 at 2 months after the fourth dose showed seroprotection rates of 67% for PCV13 serotypes but only 19% for PPSV23-exclusive serotypes (75). However, in allo-HSCT recipients without active graft-versus-host disease at 3–9 months after transplantation, the PCV13 four-dose regimen followed by PPSV23 a month later was not effective compared with a three-dose PCV13 regimen followed by PPSV23 7 months later in terms of overall IgG responses at 5 months after the PPSV23 dose (76). **Table 5** lists key findings from various studies on the immune responses and safety of pneumococcal vaccinations in patients undergoing HSCT (66–79). Across the studies, PV was well tolerated in HSCT recipients, with no serious adverse reactions related to the vaccine use (66–68, 71, 76). **Table 6** lists the optimal timing of pneumococcal vaccinations after HSCT as per guidelines (55, 60, 63, 65, 83–85).

**Table 6:** Optimal timing of pneumococcal vaccinations after HSCT as per guidelines (55, 60, 63, 65, 83–85).

NCCN (55)	After allogeneic or autologous HSCT: 3–4 doses of pneumococcal vaccination 3–6 months after HSCT • “If PCV20 is used, the first 3Ds are generally administered 1–2 months apart, with the fourth dose administered 6 months after the third dose”. • “If PCV15 is used, 3Ds should be administered, followed by PPSV23 6–12 months after the primary series”.
National Guidelines for Hematopoietic Cell Transplantation, ICMR (83)	^††^Allogeneic transplant: • PCV13: “12 months after HSCT” • PPSV23: “10 months after PCV13” Autologous transplant: • PCV13: “6 months after HSCT” • PPSV23: “10 months after PCV13”
ECIL (84)	After allogeneic or autologous HSCT: • PCV13: “3Ds, at 1-month intervals, 3 months after the transplant” • PPSV23: “1D (≥8 weeks after the last PCV) 12 months after transplant in the absence of chronic GVHD” Note: “If there is chronic GVHD, a fourth dose of PCV at 6 months after the third PCV dose The same schedule for children and adults”.
IMA (60)	PCV: “3Ds at 3–6 months after HSCT” PPSV23: “1D at 12 months after HSCT”
AGIHO (65) AGIHO (85)	Autologous HSCT: “3Ds of PCV13, each 4–6 weeks apart, starting 3–6 months after transplant, followed by 1D of PPSV23 after ≥8 weeks”.
	After allogeneic HSCT (3–6 months): “3Ds of PCV13, 4 weeks apart, and PPSV23 1 year after transplant”
IDSA (63)	After HSCT: • PCV13: “3Ds to adults and children 3–6 months after HSCT” • PPSV23: “1D, 12 months after HSCT in the absence of chronic GVHD”

^††^To be initiated once the patient is off immunosuppression (1 month) with no signs of GVHD.

1D, One dose; 3Ds, Three doses; AGIHO, Infectious Diseases Working Party of the German Society for Hematology and Medical Oncology; ECIL, European Conference on Infections in Leukemia; GVHD, Graft-versus-host disease; HSCT, Hematopoietic stem cell transplantation; ICMR, Indian Council of Medical Research; IDSA, Infectious Diseases Society of America; IMA, Indian Medical Association; NCCN, National Comprehensive Cancer Network; PCV, Pneumococcal conjugate vaccine; PPSV, Pneumococcal polysaccharide vaccine.

#### 3.2.7 Consensus recommendations

Individuals with HMs are at an increased risk of pneumococcal disease due to underlying malignancy and subsequent immunosuppressive anticancer therapy. The experts unanimously agreed that pneumococcal vaccinations are important for patients with HMs (high consensus). PCVs induce a T-cell–dependent immune response and provide adequate protection against pneumococcal disease in patients with HMs (high consensus). Experts concurred that PCVs have an acceptable safety and tolerability profile in patients with HMs (high consensus). Priming with PCV enhances the response to PPSV23 in patients with HMs. (high consensus). Experts recommended vaccination with PCV first followed by PPSV23 8 weeks later as the optimum strategy to sequence pneumococcal vaccinations in Indian patients with HMs following assessment of their immune status (high consensus). PCV is beneficial and can be strongly recommended in patients with CLL, MM, and patients undergoing HSCT (high consensus for all; GOR: ++ for all). In patients with CLL, the experts recommended administering PCV13 as soon as possible following diagnosis or at least 2 weeks before ChT (high consensus). For patients with MM, PCV13 can be considered in ND patients or at least 2 weeks before ChT (high consensus). Based on moderate consensus, PCV13 may also be administered during maintenance in this patient population. In patients with HM undergoing HSCT, PCV13 can be administered 6–12 months after the transplant (high consensus). For patients undergoing splenectomy, the experts strongly recommend administering PCV13 at least 2 weeks before the planned procedure or 14 days after the surgery if preoperative vaccination is not possible (high consensus).

Children with ALL would benefit from systematic revaccination with PCV after ChT. PCV13 should be administered to pediatric patients with ALL 6 months after the completion of ChT. PCV13 may also be administered during maintenance therapy in this patient population (moderate consensus). There are insufficient data to consistently recommend pneumococcal vaccination to all adult patients with ALL. A case-by-case evaluation and decision on vaccination are necessary. The experts suggested administering PCV13 3–6 months after the end of ChT in this subset of the patient population (high consensus). In patients with AML, there are insufficient data to recommend pneumococcal vaccination. A case-by-case evaluation and decision on vaccination are necessary. Experts suggested that PCV13 can be administered 3–6 months after the end of ChT in patients with AML (moderate consensus). Furthermore, enough data to consistently recommend pneumococcal vaccination to all patients with lymphoma are not available. Due to insufficient data, the experts suggested a case-by-case evaluation and decision on vaccination for this patient population. Experts suggested the administration of PCV13 before treatment in ND patients or at least 2 weeks before ChT (high consensus). PCV13 may also be administered during maintenance in this patient population (moderate consensus).

### 3.3 Barriers to pneumococcal vaccination in the hemato-oncology clinical setting in India

Globally, barriers to pneumococcal vaccination uptake include concerns about vaccine efficacy, lack of awareness of the disease complications, and vaccination schemes (86, 87). The expert panel identified a lack of awareness about pneumococcal disease, its risk factors or vaccine availability, and lack of recommendation from healthcare practitioners (HCPs) for vaccination as the most common patient-level barriers to pneumococcal vaccination in the hemato-oncology clinical setting in India. At the HCP level, the most common perceived barriers were a lack of awareness about the burden of pneumococcal disease, uncertainty about clinical data on vaccine effectiveness, and a lack of clarity regarding the timing and schedule of pneumococcal vaccination in complex treatment schedules. Educating HCPs about vaccine guidelines and providing patient counseling about the risk of pneumococcal disease in HMs and the importance of receiving early vaccination can play an important role in reducing the risk of serious infections and adverse events among this vulnerable population.

## 4 Discussion

Pneumococcal diseases are a significant health concern for patients with HMs. Serious pneumococcal infections include pneumonia, meningitis, and febrile bacteremia; however, otitis media and sinusitis are less serious manifestations (88, 89). Two recent systematic reviews reported the effectiveness of pneumococcal vaccination for protection against vaccine type-(PCV13 type or PPSV23 type) IPD and CAP due to *S. pneumoniae* in adults (90, 91). In addition to reducing the medical burden, pneumococcal vaccination may provide economic benefits by lowering the risk of hospitalization due to its contribution to preventing pneumococcal disease and adverse outcomes (5, 92, 93). In India, PCV was introduced into the pediatric national immunization schedule recently in 2017 (94). Thus, the concept of herd immunity is not applicable in India. Currently, PCV10 and PCV13 are licensed and available in the private sector. PCV13 protects against three additional prevalent serotypes (3, 6A, and 19A) not included in PCV10 (95–97). Considering this, the experts recommended vaccination with PCV13 first, followed by PPSV23, as the optimum strategy to sequence pneumococcal vaccinations in patients with HMs in India. Furthermore, the expert panel strongly recommended PCV13 in CLL, MM, and patients undergoing HSCT. When higher-valency PCV20 and PCV15 become available in India, they will replace the existing PCV13 for adult vaccination. Considering the current evidence of the risk of pneumococcal disease in patients with HMs and the recommendations and consensus for the use of PCV13 and PPSV23 in these patients, it is important to consider how PCV15 and PCV20 can be utilized to extend the benefit of protection against pneumococcal disease in this high-risk patient population in Indian settings.

### 4.1 Strengths and limitations of consensus process

#### 4.1.1 Strengths

Our modified Delphi study methodology represents a rigorous synthesis of expert opinions. The expert committee was formed without any selection bias. The survey was designed after an in-depth literature review to increase the study’s rigor and ensure efficient responses. Anonymity was maintained during the survey to encourage panel members to provide honest and unbiased feedback. All experts actively participated in the consensus process. The differences in opinions were also discussed during the meeting. The diverse panel helped achieve a broader perspective and generalization of consensus. These evidence-based practical consensus recommendations can guide practicing clinicians and healthcare professionals nationwide in making informed decisions about vaccine sequencing, specific patient profiles that can benefit from pneumococcal vaccination, and optimal timing of vaccination in patients with HMs.

#### 4.1.2 Study limitation

This consensus process did not include any patient opinions or perspectives.

## 5 Conclusion

Preventing pneumococcal disease is paramount for high-risk individuals, and administering PV should be an important aspect of clinical care. The vaccine type and timing of vaccinations must be carefully chosen to allow for optimal immunization in patients with HMs. Prevention of pneumococcal infections by vaccination has been evaluated and found to be a viable strategy. Priming with PCV enhances the response to PPSV23 in patients with HMs. The experts recommended vaccination with PCV13 first, followed by PPSV23 8 weeks later, as the optimum strategy to sequence pneumococcal vaccinations in patients with HMs in Indian settings. In patients with CLL and MM, the experts recommended administering PCV13 as soon as possible following diagnosis or at least 2 weeks before ChT. In patients with HM undergoing HSCT, PCV13 can be administered 6–12 months after the transplant. PCV13 should be administered to pediatric patients for ALL 6 months after the completion of ChT or during maintenance. For patients undergoing splenectomy, the experts strongly recommend administering PCV13 at least 2 weeks before the planned procedure or 14 days after the surgery if preoperative vaccination is not possible. The current state of evidence is inadequate to consistently recommend pneumococcal vaccination to all patients with lymphoma, AML, and adults with ALL. The decision to administer PCV13 preceding PPSV23 should be taken jointly by the HCP and the patient on a case-by-case basis after carefully discussing the pros and cons.
