# Supplementary material for: Preventing pneumococcal infections in patients with hematological malignancies: a review of evidence and recommendations based on modified Delphi consensus
Source: Front Oncol. 2025 May 1;15:1546641. doi: 10.3389/fonc.2025.1546641 (PMC12078153; doi:10.3389/fonc.2025.1546641)
Supplement: Supplementary file 1 [file Table1.docx]

**Supplementary file**

**Supplementary Table S1.** Immune responses and safety of pneumococcal vaccinations in children with ALL (35–37)

| **Author and year** | **Study design and patient population** | **Type of pneumococcal vaccine administered** | **Key results** |
| --- | --- | --- | --- |
| Bate J *et al.* 2020  (35) | Nonrandomized, open-label study  Pediatric patients with ALL on maintenance treatment or completed treatment within the last 6 months | A single dose of PCV13 at the following time points:  Group 1 (n=39): During maintenance ChT  Group 2 (n=40): 4 weeks after the last oral maintenance ChT  Group 3 (n=39): 6 months after the last oral maintenance ChT | Protective response rates   - 12 months after vaccination: 0%, 37.9%, and 43.3% in groups 1, 2, and 3, respectively   No pneumococcal disease during the study period |
| Dorval S *et al.* 2021  (36) | Retrospective observational study  Children treated for ALL fully immunized with three PCV doses before ALL diagnosis | Group 1: One PCV13 booster dose during maintenance and another dose after ChT (n=32)  Group 2: One PCV13 booster dose after ChT (n=39) | - Seroprotection at the end of ChT: 53.1% in group 1 vs. 25.6% in group 2 (p=0.018) - Seroprotection post-ChT PCV13 booster: 96.9% in group 1 and 100% in group 2 |
| Top KA *et al.* 2020  (37) | Prospective clinical trial  Children with ALL 4–12 months after completing ChT (N=74)  Immunocompetent children as controls (N=78) | Patients with ALL received PCV13 followed by PPSV23 after 2 months | Immune responses after vaccination:   - Seroprotective IgG levels against all PCV13 serotypes in 61% of patients at 2 months and in 34% of patients by 12 months after PCV13 - Increased antibody levels to PPSV23 serotypes (p<0.001)   Safety   - No SAEs |

ALL: Acute lymphoblastic leukemia; ChT: Chemotherapy; IgG: Immunoglobulin G; PCV: Pneumococcal conjugate vaccine; PPSV: Pneumococcal polysaccharide vaccine; SAE: Serious adverse event.

**Supplementary Table S2.** Immune responses and safety of pneumococcal vaccinations in patients with lymphoma (49–54)

| **Author and year** | **Study design and patient population** | **Type of pneumococcal vaccine administered** | **Key results** |
| --- | --- | --- | --- |
| Grimfors G *et al.* 1990  (49) | Longitudinal study  Splenectomized patients  HD (N=41)  NHL (N=25)  Controls: Splenectomy for trauma (N=33) | PPSV  (14 or 23 serotypes) | Overall efficacy:   - No differences in the response to vaccination between patients vs. controls   Efficacy among patients with HD (2 weeks after vaccination)   - Better response in patients vaccinated before vs. after splenectomy and treatment (p<0.05)   Responses after 2 weeks vs. at 3 years   - Patients with HD: 32% vs. 7%; Controls: 48% vs. 21%   Safety   - No severe adverse reactions to vaccination |
| Petrasch S *et al.* 1997  (50) | Splenectomized patients with B-cell NHL (N=11)  Controls: Splenectomy for other reasons (N=7) | PPSV23 before lymphoma treatment and splenectomy | Responses to initial immunization:   - Rise in antibody titers in 45.4% of patients with NHL vs. 57.1% of controls   Responses to booster immunization in patients with NHL:   - No significant increase in antibody titers   Safety:   - Mild adverse reactions to vaccination |
| Landgren O *et al.* 2004  (51) | Prospective study  Splenectomized patients with HD (N=208)  Controls: Splenectomy for trauma (N=28) and immune-mediated cytopenia (N=75) | PPSV23 (patients revaccinated based on individual antibody levels) | Antibody response:   - Significant response to primary vaccination and two revaccination occasions   Pneumococcal infections among clinically monitored patients with HD:   - Ten OPSIs in seven patients during the study period   Safety:   - No severe adverse reactions to vaccinations |
| Cherif H *et al.* 2006  (52) | Prospective study  Splenectomized patients with hematological disorders (N=76) | PPSV23 (patients revaccinated based on individual antibody levels) | Antibody response   - Poor response in 28% of patients, of whom 95% showed no improvements after revaccination   Clinical characteristics of poor responders   - Significant difference in median age at vaccination between poor vs. good responders (57 vs. 48 years, p=0.0006) - Pneumococcal infections after vaccination - Five episodes in three patients, all in poor responders   Safety   - No severe reactions to vaccinations |
| Molrine DC *et al*. 1995  (53) | Previously treated patients with HD without relapse or second tumors (N=128)  Healthy immunized controls (N=19) | Patients randomly assigned to PPSV23 (n=58) or PCV7 (n=70) | PPSV23 (patients vs. controls)   - IgG GMC was similar between patients and controls   PCV7 (patients vs. controls)   - IgG GMC was lower in patients vs. controls (p<0.05)   PCV7 vs. PPSV23 in patients   - IgG GMCs were lower in patients given PCV7 vs. PPSV23 (p=0.0005) |
| Lee D *et al*. 2023  (54) | Retrospective study  Patients with relapsed or refractory large  B-cell lymphoma who underwent CAR-T (N=148) | PCV13 at 3, 6, or 12 months after CAR-T | - Decrease in IgG titers from day+90 to day+180 (p=0.03) |

CAR-T: Chimeric antigen receptor-modified T cell therapy; GMC: Geometric mean concentration; HD: Hodgkin disease; IgG: Immunoglobulin G; NHL: Non-Hodgkin lymphoma; OPSI: Overwhelming post-splenectomy infection; PCV: Pneumococcal conjugate vaccine; PPSV: Pneumococcal polysaccharide vaccine.
